# Ohio College of Dental Surgery

**Published:** 1850-10

**Authors:** 


*Ohio College of Dental Surgery.*—We are gratified to learn from the Sixth Annual Announcement, that the vacancies in this institution have been filled. The following are the names of the gentlemen, who, under the present organization, compose the Faculty, namely: James Taylor, M. D., D. D. S., Professor of Practical Dentistry, and Pharmacy; Thomas Wood, M. D., Professor of Anatomy and Physiology; George Mendenhall, M. D., Professor of Pathology and Therapeutics; A. M. Leslie, D. D. S., Professor of Mechanical Dentistry; and William A. King, D. D. S., Demonstrator of Mechanical Dentistry and Assistant Surgeon in the infirmary.

With Dr. Taylor, we have been personally acquainted for many years, and know him to be zealously devoted to the interests of the profession and the advancement of dental surgery. We doubt whether a more competent person could have been obtained for the chair which he occupies in the institution. Drs. Wood and Mendenhall are too favorably known in the medical profession to require commendation; and we doubt not that Drs. Leslie and King are well qualified to teach the branches to which they have been assigned.

With the institution of colleges for the education of young gentlemen for the practice of dental surgery, commenced a new era in the history of the profession. Previously to this time it was supposed, except by the educated and better informed practitioners, that a sufficient amount of knowledge might be obtained in a few days or weeks, or months at most, in the mechanical work-shop or laboratory of a dentist; and hence, the ranks of the profession had, for years, been rapidly filling with ignorant and illiterate men, until the very name, in many parts of the country, had become a by-word of reproach.

But, although this state of things is gradually passing away, and although a higher standard of qualification is beginning to be regarded as necessary to successful practice, years will still have to elapse before a college of dental surgery will be sufficiently patronised to afford professors a fair remuneration for the labor and time they must necessarily bestow in teaching, or to refund the money which ought to be expended in the procurement of the necessary facilities for it, and defray the current expenses of such an institution. Those, therefore, who are at this time engaged in teaching, must be influenced by higher motives than present pecuniary reward, if they would not be disappointed in their hopes and expectations. But we believe the time will ultimately arrive, when the demand for dental collegiate education will be amply sufficient to sustain three or four colleges in the United States.

